# Conductive textiles prepared by spray coating of water-based graphene dispersions†

**DOI:** 10.1039/c9ra09164e

**Published:** 2020-01-13

**Authors:** Archana Samanta, Romain Bordes

**Affiliations:** Department of Chemical Engineering, Chalmers University of Technology Gothenburg Sweden bordes@chalmers.se

## Abstract

In the development of embedding devices on textiles like sensors and detectors the controlled formation of a conductive coating remains a critical point. Although there are several approaches for imparting conductivity to any textile, the challenges remain in balancing the practical aspects of the coating procedure that affect the conductivity with the associated mechanical properties of the textile along with a feasible economic viability of the process. In this research we developed an approach to deposit uniform conductive graphene surface coatings on polyester (PET) fabric using graphene oxide (GO) particles. Instead of using pre-reduced graphene oxide (rGO), an approach of coating an aqueous dispersion of hydrophilic GO particles was adopted. Stable aqueous dispersions of GO were formulated, and the dispersion properties were characterized using DLS and zeta potential before coating them onto the PET textiles. This approach not only helped in developing an aqueous coating technique but also helped in avoiding the need of any organic solvents which might have been required for coating hydrophobic rGO moieties onto the textile substrates. The uniformity of the coating was analyzed using scanning electron microscopy (SEM). Later, the GO coated textiles were reduced *via* thermal and chemical approaches and their effects on the conductive and mechanical properties of the fabric were assessed and compared. The reduction efficacy was analyzed and compared using XPS. The conductivity and water adsorption properties were correlated to the uniformity and retainment of rGO on the surface of the conductive textiles.

## Abstract 2

Effect of coating technique on surface properties and conductivity of PET textiles.

## Graphical abstract

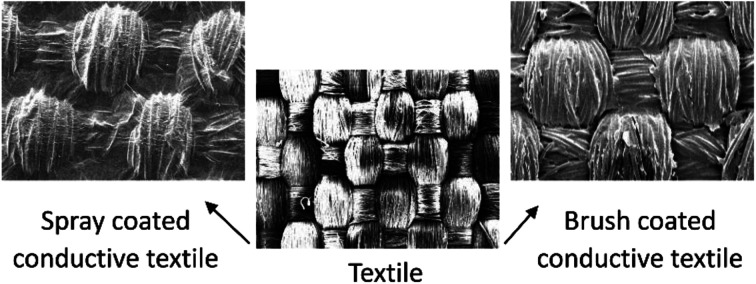

## Introduction

Smart or intelligent textiles have gained importance in the field of flexible electronics owing to their specific properties of low bending rigidity, stretchability, ease of handling, light weight and breathability.^1–3^ Apart from their general usages in electronics,^4^ embedded fashionable garments^5^ and photo luminescent textiles,^6^ these textiles are also used in various high-end applications like piezo-electric sensors,^7^ wearable batteries,^8^ EMI shields,^9^ osmotic membranes^10^ and others. Ease of imparting conductivity and its long-term retainment on the textiles are key features dictating the performance of such finished textiles.

In general, fabrication of conductive textiles can be done through two approaches; (a) weaving or knitting a textile with conductive yarn of copper/silver/carbon fiber^11^ or (b) depositing conductive particles onto a woven or knitted fabric *via* suitable coating process.^12^ The second process is more commonly considered because of economic viability and ease of processing. Micro or nano dimensional particles of silver,^13^ gold,^14^ carbon nano tubes,^15^ carbon black,^16^ graphite^17^*etc.*, have been used for imparting conductive coatings. Among these class of particles, graphene has been of interest because of its high thermal conductivity (∼3000 W m^−1^ K^−1^) and high electron mobility (15 000 cm^2^ V^−1^ s^−1^) along with comparatively high Young's modulus of ∼1.0 TPa, and high specific surface area owing to its two-dimensional nano structures with sp^2^ bonded conjugated carbon system.^18–20^ Because of these properties graphene based coatings have attracted interest for usage in the fields of photosensors,^21^ gas sensors,^22^ wearable electronics^23^ and others.^24^

However, realizing a uniform graphene coating remains a challenge in practice, as graphene cannot be handled as other conductive particles that are typically dispersible in a solvent, quite often water. This latter feature enables application of conductive nanoparticles by dip coating process,^25^ where the solvent is later removed by evaporation assisted with heat treatment. Brush coating,^26^ screen printing,^27^ electrochemical deposition^28^ techniques are also used for producing conductive coatings. However, their usage is limited due to the multi-step process and difficulty in scaling up. Furthermore, these treatments have poor environmental footprint as they consume large amount of water. One alternative to graphene is indeed graphene oxide (GO). GO differs from graphene in the sense that it has several oxygen functionalities at the edge and basal planes, which enables forming a stable aqueous dispersion. These groups increase the material hydrophilicity and enable water dispersibility. But at the same time they interrupt the sp^2^ carbon conjugation, resulting in meager conductivity;^29^ an issue that is typically remediated by *in situ* reduction. Thermal reduction and chemical reduction are the two mainly used process for reducing graphene oxide to its conductive form.^30^ Thermal reduction process is the simplest and most convenient technique to form highly conductive reduced graphene oxide (rGO).^31^ Alternatively, chemicals can be applied to GO once coated onto the desired substrate, through the addition of a suitable reductant. The sample are then washed thoroughly to get rid of the excess of reductant. Various chemical agents such as sodium hydrosulphite,^32^ hydrazine,^33^ sodium borohydride,^34^ oxalic acid,^35^ hydriodic acid^36^ and others have been used for chemically reducing graphene oxide. Green alternatives, such as glucose,^37^ citric acid,^19^ ascorbic acid,^38^ bovine serum albumin,^39^ polyphenols^40^ (present in green tea and caffeic acid) *etc.* are being used either solely or in combination with other chemical reductants or temperature to reduce graphene oxide.^41^

An investigation of the recent studies involving applications of graphene for conductive textiles, showed that most of these processes emphasized on chemical reduction *via* dip coating process, for example coating of graphene oxide and AgNO_3_ onto PET textile and its subsequent reduction with hydrazine^42^ or in another study Sahito *et al.* coated positively charged cotton fabrics with GO and reduced it using hydrazine hydrate vapors.^43^ Berendjchi *et al.* immersed the PET fabrics in GO dispersion at 70 °C for 1 h and then cured at 80 °C for 60 min. These samples were then immersed in Na_2_S_2_O_4_ solution at 90 °C for 60 min. And were then coated with poly(pyrrole) for better conductivity.^44^ In another study polypropylene non-woven webs were coated with GO dispersions in DMF which was then reduced using hydroiodic acid. Generally, a combination of dip coating with assisted chemical reduction process is adopted to fabricate conductive textiles.

In the present study we suggest an alternative that capitalizes on spray coating of dispersion of graphene oxide. GO coated textiles are reduced using thermal reduction, chemical reduction using NaBH_4_ and green reduction approach using l-ascorbic acid which is a nontoxic mild reducing agent^45^ and a comparative analysis of the reduction methods affecting their properties is studied in details. The motivation is to develop a continuous, energy efficient, economically viable and environmentally friendly coating methodology which can be easily adopted easily by textile industries for large scale fabrication of conductive coatings.

## Experimental

### Materials

Plain woven 100% polyester fabrics was kindly supplied by FOV fabrics, Sweden, and was used as received. Graphene oxide powder (with reported particle size < 70 μm and BET surface area of 400 m^2^ g^−1^) was procured from Avanzare, Spain. l-Ascorbic acid (99%) and sodium borohydride (98%) were procured from Sigma-Aldrich, Sweden. A nonionic surfactant, Ethylan 1008, was procured from AkzoNobel, Sweden. MilliQ water was obtained from a Millipore system.

### Coating graphene oxide nanosheets on PET textiles

Aqueous dispersions of graphene oxide (ranging from 0.01 to 0.5 weight% concentrations) were prepared in deionized water by sonication using Sonic vibra-cell processor VC505 operating at 75% amplitude for 10 minutes. These dispersions were then immediately sprayed onto a 10 cm × 10 cm of PET textile and then dried at room temperature for 24 h. Formulations with a 1 : 1 weight ratio of GO and Ethylan 1008 were also applied with same concentration and were sprayed to similar dimensions of PET textiles. Samples with surfactant were washed thoroughly with deionized water before annealing. For comparing the efficacy of spray coating, mechanical brushing was carried out using a paint brush onto the same dimension of PET textile with similar formulations.

### Reduction of graphene oxide coated fabrics

For thermal reduction, the dried coated samples were annealed at 200 °C for a duration of 2 h. This time was found to be optimal to achieve the maximum conductivity, from a preliminary study. For chemical reduction, the samples were immersed in 10 weight% concentrated solution of ascorbic acid or NaBH_4_ at room temperature for a period of 48 h. Similar process is detailed in the literature.^38,45,46^ Samples were then washed by dipping them in deionized water 3 times after the reduction duration and then were dried at room temperature for 24 hours.

### Characterization

Scanning electron microcopy (SEM) was used to observe the surface topography of the samples using a JEOL 7800F Prime instrument. Samples were pre-coated with palladium by sputtering at an accelerating voltage of 10 kV before observation under SEM.

Conductivity surface conductivity of the samples was determined using a homemade 4 probe devices. Samples were placed on a flat insulating surface and the probes were contacted with the samples to get the readings on a digital screen. Sample dimensions were 2 cm × 5 cm. Surface conductivity (mS m^−1^) was calculated by dividing length of the tested sample with the product of measured resistance and area of cross section of the textile which was around 1.4 × 10^−6^ m^2^.

Dynamic light scattering was performed on Beckman Coulter, N4 plus sub-micron size analyzer to determine the particle size of the dispersions after sonication. Zeta potential was measured using a Zeta PALS zeta potential analyzer from Brookhaven instrument corporation with a red He–Ne laser (*λ* = 633 nm). Contact angle was measured using an Attension Theta optical tensiometer (Biolin, Finland). A drop of 2-5μl of water was applied to measure the contact angle and the time of absorption for the dried rGO samples.

X-ray Photoelectron Spectroscopy (XPS) was carried out to evaluate the graphene coating and chemical reduction. XPS was conducted using a PHI VersaProbe III Scanning XPS Microprobe from Physical Electronics; X-ray excitation was provided by Al Kα radiation with photon energy *hν* = 1486.6 eV. A sample area of approximately 400 × 500 μm was scanned, with an indicative information depth of about 4–5 nm. XPS measured the number of electrons and their corresponding kinetic energies which were released when the textile was irradiated with the X-ray beam. These information was then corelated to determine the binding energy spectra and composition of the coating at parts per thousand range.^47^

Tensile properties were measured using Instron 5565 instrument. Samples were preconditioned at 65% relative humidity, 20 °C for 12 hours before testing. A strain rate of 500 mm min^−1^ was applied to fracture the samples that had a 2 cm × 5 cm as dimension. An average of 5 readings is reported.

## Results and discussion

### Dispersion of the GO sheets

Aqueous dispersions of GO were formulated with and without surfactant for comparison of the dispersion stability and determination of coating uniformity. A 1 : 1 (wt/wt) dispersion of nonionic surfactant Ethylan 1008 and GO was formulated in deionized water aided with sonication. Starting with 0.01 wt%, the concentration of GO was increased to 0.5 wt%. The idea behind the usage of lower concentration of GO was to form a stable homogenous dispersion with good sprayability which could then form a uniform micron-thick layer deposit on the textiles without hampering the stiffness and handle of the textiles. Also 0.5% was the limiting dispersion concentration in our study with the selected GO beyond which the colloidal stability of the formulation was affected.

DLS was used to identify the average size of the GO aggregates in water. Though the GO are present in form of flakes, the value from DLS provided an indication of the overall GO aggregated dimensions in the dispersions and the values are reported in Table 1. As can be seen, the average particle size increases slightly with increase in GO concentration. Further, usage of 1 : 1 weight fraction of the surfactant with respect to GO resulted in reducing the particle size by preventing their aggregation. However, the trend is similar in both cases, *i.e.* increase in GO concentration results in formation of larger particles in the dispersions. This can be caused by remnants of aggregated GO particles at higher GO contents as the dispersion and sonication period was kept constant in all cases. As the presence of Ethylan 1008 helped in achieving lower sizes of GO dispersions, it was considered that in these dispersions presence of smaller GO moieties can result in uniform film coatings compared to the non-surfactant ones with same GO content and can potentially result in better conductivity of GO sheets by imparting better connectivity in the final product.

**Table 1:** Particle size and zeta potential of aqueous GO dispersions

Sl no.	Concentration of GO (wt/wt%) in aqueous dispersion	Surfactant used	Particle size DLS (nm)
1	0.01	—	406 ± 4
2	0.1	—	471 ± 9
3	0.5	—	512 ± 14
4	0.01	Ethylan 1008	385 ± 7
5	0.1	Ethylan 1008	418 ± 9
6	0.5	Ethylan 1008	484 ± 12

Zeta potential of the GO particles was measured to check if their oxidation degree would ensure electrostatic stabilization dispersion. Irrespective of the concentration, the zeta potential remains constant with values −37 mV and −40 mV for GO dispersions with and without surfactant respectively, which provided sufficient stabilization. As expected, the use of non-ionic surfactant did not influence the zeta potential values, since the surfactant is meant to provide steric stabilization. As the surfactant free dispersions had reasonable colloidal stability compared to the ethylan GO dispersions, an attempt was also made to study and compare their film formation tendency and conductive properties.

### GO film formation on the textiles

Dip coating process is usually used in the textile industries for bulk fabrication of coated textiles. However, this process utilizes large amount of water which is then removed by application of heat/energy and also leaves excess of material that needs to be recovered. Hence, spray coating approach was adopted as an alternative finishing process. For comparison, brush coating was also tested.

The purpose of this study was to utilize the hydrophilic characteristic of GO to form a direct aqueous dispersion, coat them to the textiles and then reduce the GO layer. This is because of better dispersibility of GO compared to its reduced form in aqueous media. Therefore, we sprayed 1 ml of GO dispersions with various GO concentrations onto a 10 × 10 cm^2^ of textiles and the dry weight increase was calculated from the weight change in the dried coated the samples. Summary of the samples details is given in Table 2.

**Table 2:** Formulation details, conductivity and contact angle values of coatings

Code	Conc. of GO wt%/application process	Weight increase^a^ (%)	Vol. of spray (ml) per 10 × 10 cm^2^	Reduction process	Surface conductivity (mS m^−1^)	Contact angle of the fabric^c^ (°)/time of absorption (s)
SE1	0.01 spray, Ethylan 1008^b^	0.02	1	200 °C, 2 h	—c	65° ± 11, 95 ± 14 s
SE2	1.1 spray, Ethylan 1008^b^	0.17 ± 0.02	1	200 °C, 2 h	—c	68° ± 7, 135 ± 17 s
SE3	0.5 spray, Ethylan 1008^b^	0.92 ± 0.04	1	200 °C, 2 h	0.31 ± 0.07	74° ± 8, 152 ± 11 s
SE4	0.5 spray, Ethylan 1008^b^	2.3 ± 0.06	3	200 °C, 2 h	0.37 ± 0.05	76° ± 10, 155 ± 8 s
SE5	0.5 spray, Ethylan 1008^b^	4.5 ± 0.05	5	200 °C, 2 h	0.45 ± 0.11	76° ± 14, 159 ± 15 s
S1	0.01 spray	0.01 ± 0.01	1	200 °C, 2 h	—c	69° ± 7, 110 ± 14 s
S2	0.1 spray	0.07 ± 0.02	1	200 °C, 2 h	0.37 ± 0.09	71° ± 8, 158 ± 10 s
S3	0.5 spray	0.37 ± 0.08	1	200 °C, 2 h	0.48 ± 0.10	71° ± 3, 900 ± 10 s
S4	0.5 spray	1.10 ± 0.07	3	200 °C, 2 h	0.69 ± 0.07	75° ± 2, 1200 ± 7 s
S5	0.5 spray	1.83 ± 0.44	5	200 °C, 2 h	2.38 ± 0.11	79° ± 8, 1500 ± 12 s
SA1	0.5 spray^b^	0.37 ± 0.95	1	l-Ascorbic acid	0.48 ± 0.11	70° ± 5, 120 ± 9 s
SA2	0.5 spray^b^	1.10 ± 0.75	3	l-Ascorbic acid	0.55 ± 0.08	73° ± 4, 660 ± 12 s
SA3	0.5 spray^b^	1.83 ± 0.61	5	l-Ascorbic acid	0.83 ± 0.09	74° ± 2, 785 ± 12 s
SN1	0.5 spray^b^	0.37 ± 0.89	1	NaBH_4_	0.37 ± 0.06	68° ± 3, 109 ± 7 s
SN2	0.5 spray^b^	1.10 ± 0.94	3	NaBH_4_	0.48 ± 0.08	77° ± 3, 133 ± 9 s
SN3	0.5 spray^b^	1.83 ± 0.45	5	NaBH_4_	0.69 ± 0.07	64° ± 4, 186 ± 17 s
B1	0.5 brush	0.37 ± 0.12	1	200 °C, 2 h	0.41 ± 0.07	65° ± 8, 720 ± 10 s
B2	0.5 brush	1.10 ± 0.08	3	200 °C, 2 h	0.53 ± 0.04	66° ± 5, 840 ± 7 s
B3	0.5 brush	1.83 ± 0.52	5	200 °C, 2 h	1.28 ± 0.09	72° ± 7, 1055 ± 15 s
PETN	PET neat^c^	NA	NA	NA	c	110° ± 2, 62 ± 4 s

a % Dry weight increase after coating with GO.

b Samples containing Ethylan 1008, ascorbic and NaBH_4_ were washed thoroughly with deionized water after the drying of the sprayed formulations.

c Surface resistance was too high to be measured by the instrument.

**Fig. 1:**
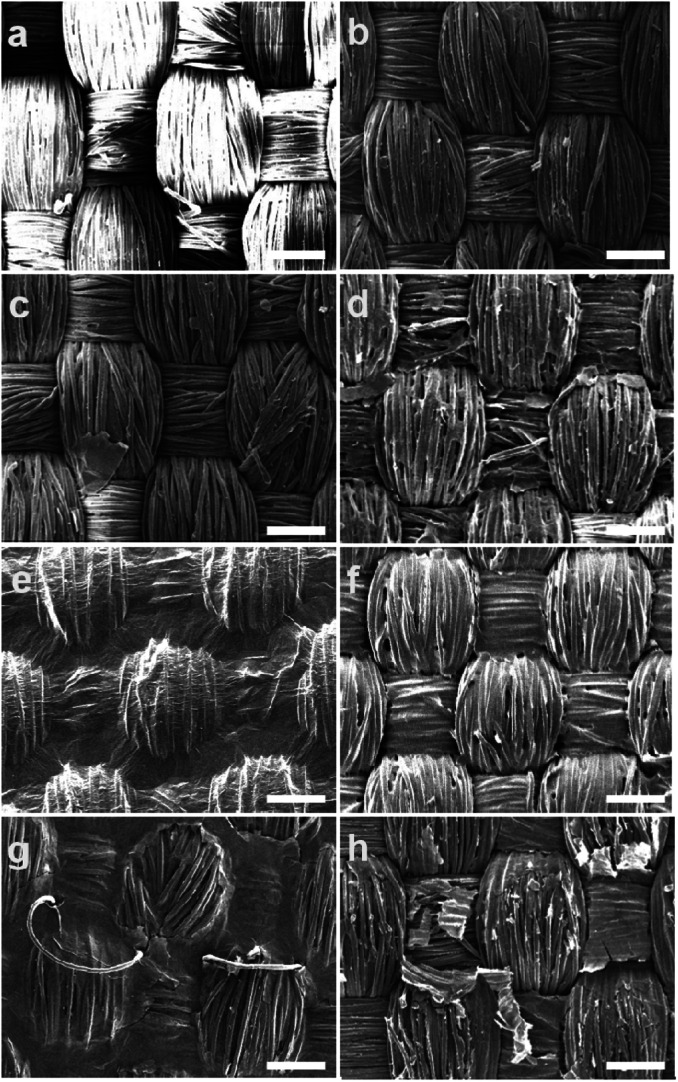
SEM images of surface topography of GO spray coated samples. (a) Neat PET (b) S1 0.01% GO (c) S2, 0.1% GO (d) S3, 0.5% GO (e) S5, 1.83% GO (non-annealed) no surfactant spray coated (f) B3, 1.83% GO (non-annealed) brush coated (g) SE5 1.83% GO (non-annealed) with Ethylan 1008 spray coated (h) SE5 GO (non-annealed) with Ethylan 1008 washed before reduction. The white bar indicates a scale of 100 μm.

Two sets of textiles with and without surfactant were coated with similar concentration of GO as depicted in Table 2. In both cases, textiles coated with 0.01 wt% of GO dispersion showed poor interconnectivity of GO layer on the surface, as shown by the SEM images, Fig. 1. A volume of 1 ml was kept constant as this volume was found to provide enough wettability of fabric coating. GO concentration was increased to 0.1 wt% and then to 0.5 wt%. Application of 0.5 wt% resulted in better coverage of the fabric but non-continuity of coating was still prevalent. Above 0.5 wt%, the dispersibility of GO was affected and sedimentation occurred. To overcome this the volume of the formulation was increased from 1 ml to 3 ml and then to 5 ml per 10 × 10 cm of the textile to increase dry weight% values to from 0.37% to 1.10% and 1.83%, respectively. This resulted in the formation of a continuous uniform coating of GO on the surface of the fabric as shown by SEM. With 1.83% a well-connected GO layer was formed on the surface of the textiles even without the surfactant (Fig. 1e and g). Samples containing surfactant were washed with deionized water to remove excess surfactant from the surface which otherwise would have impacted the surface conductivity. The SEM image represented in Fig. 1h, shows removal of GO layers from the surface in the washed samples, implying that though the presence of surfactant would help stabilizing the GO dispersion it could induce a loss of material upon washing.

For comparison, brush-coated samples with similar GO content and volume were prepared. SEM image of brush coated sample with highest GO content *i.e.* of 1.83% is shown Fig. 1f. As can be observed the spray coated sample has more uniform GO film as compared to the brush coated sample with same GO content. This gives an indication that during brush coating the GO distribution is not uniform, probably the GO moieties are unable to spread uniformly onto the contours of interlacement points of warp and weft by the mechanical act of brush coating.

### Reduction of the GO sheets

Three different reducing methods namely; thermal reduction, chemical reduction using NaBH_4_ and a green reductant *i.e.* ascorbic acid were used. Thermal reduction of the GO coated textiles was carried out at 200 °C for 2 h. It has been reported that around temperatures of 150–200 °C, majority of the oxygen is removed *via* desorption of CO_2_, H_2_O and CO from the GO films.^48^ Percolative electron transfer for attaining conductivity is attained by removal of oxygen from the structuring rending proximity of fused carbon ring structures resulting in formation of larger graphitic domains.^49^ Lowering of reduction temperature or time below these values did not yield good results. Ethylan 1008 coated samples were washed thoroughly with deionized water before annealing to get rid of excess surfactant which otherwise could impact negatively the conductivity of the coatings. Coated samples with same GO content without Ethylan 1008 were also prepared and annealed under similar conditions of temperature and time. A chemical approach was also taken. The GO coated samples were immersed in 10% aqueous NABH_4_ solution for 48 h at 20 °C. This method was already reported in literature.^46^ Samples were then washed with deionized water to get rid of excess NaBH_4_ and dried at 20 °C. In another approach the GO coated samples were immersed in 10 wt% of solution of ascorbic acid for 48 h. Samples were then washed with deionized water to get rid of excess ascorbic acid and dried at 20 °C. Similar process is reported in literature for reduction of GO *via*l-ascorbic acid.^45^

**Fig. 2:**
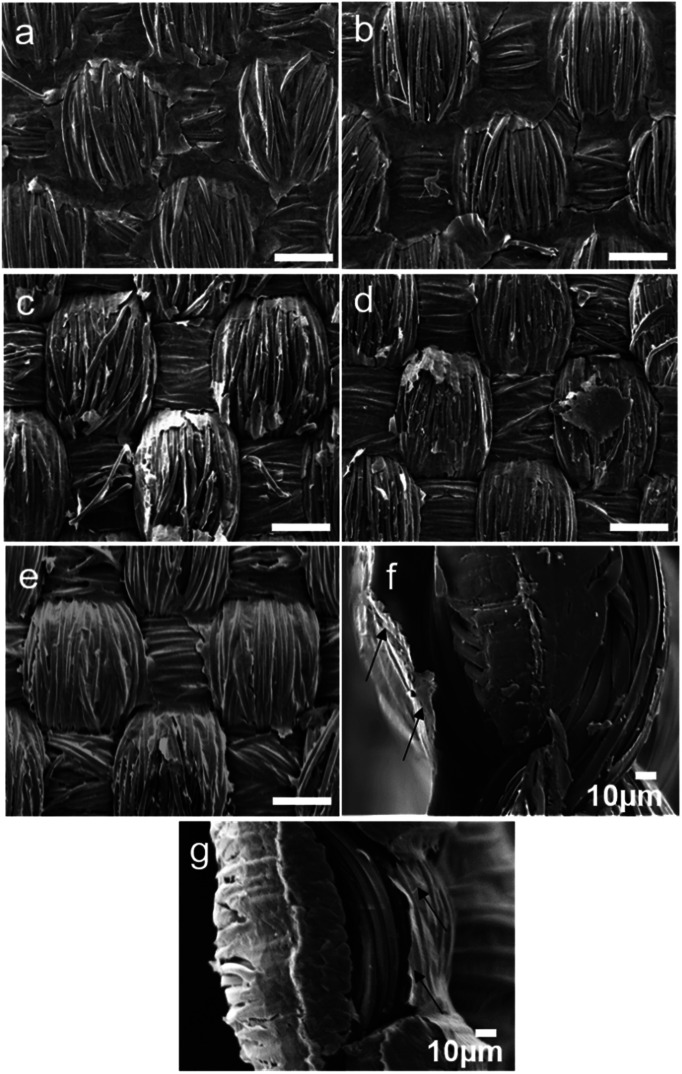
SEM images of surface topography of reduced samples. (a) S5 1.83% GO (annealed) (b) S5 1.83% GO washed after annealing (c) SN3, 1.83% GO reduced by NaBH_4_ (d) SA3, 1.83% GO reduced by l-ascorbic acid (e) B3, 1.83% GO brush coated (annealed) (f) B3 cross-section (g) S5 cross-section. The white bar indicates a scale of 100 μm, unless otherwise stated.

The efficiency of the reduction process of the GO spray coated textiles on GO coated PET samples was evaluated by XPS analysis, as reported in Table 3, while the effect of reduction on the coating integrity was evaluated by SEM, Fig. 2 and S1 (ESI†). Reduced GO forms a conjugated structure with reduced number of oxygen groups compared to its oxide state. Samples with 1.83% dry GO content, reduced *via* various aforementioned techniques which showed better surface coverage were analysed for this study. The percentage of oxygen reduces with the reduction process. GO coated samples reduced by NABH_4_ and ascorbic acid showed similar oxygen content reduction which was slightly lower than the thermally reduced PET sample. A maximum of 13% reduction of oxygen content was observed in thermally reduced GO coated PET sample. This data is also in agreement with the conductivity value which is highest with thermally annealed GO coated PET fabric at a dry GO content of 1.83%. The reduction process also affected the coating integrated and in particular through chemical approach as the samples were put under wet condition for a long period.

**Table 3:** XPS data of the samples

Sample	Atomic content (%)	
	Carbon (C)	Oxygen (O)
PET neat	67.95	30.66
PET GO thermally reduced	77.18	21.99
PET GO ascorbic acid reduced	72.90	24.82
PET GO NaBH_4_ reduced	72.89	24.11
PET coated non-reduced	68.02	31.19

### Conductivity of GO coated textiles

After reduction of GO coated textile samples, their surface conductivity was measured, as stated in Table 2. The resistance of control PET textile was very high and was outside of the measurement range of the instrument. As expected, the conductivity of GO coated samples increased with increase in GO content, irrespective of reduction process. The conductivity was also affected by the dry pick-up percentage; higher GO content resulted in better connectivity of the GO layers and hence resulted in better conductivity as shown in Fig. 2. The conductivity of thermally annealed samples coated with formulation containing surfactant was less than that of the samples coated with formulations free of surfactant. SEM images of GO-Ethylan 1008 washed unannealed samples shown in Fig. 1h, showed removal of GO layers from the surface of textiles resulting in poor connectivity of GO layers hence resulted in lower conductivity after reduction.

The continuity of the GO layers in S5 samples was retained even after annealing at 200 °C for 2 h and later washing the annealed samples (Fig. 2a and b). Annealed B3 sample showed poorly connected corners and contours, associated with brush coating process. Spray coated samples with this concentration reduced chemically by NABH_4_ and ascorbic acid are shown in Fig. 2b and c respectively. Patches of non-continuous GO regimes could be observed, which presumably result from the removal of reduced GO layer during the dip immersion and washing stages of the reduction process. This hypothesis is validated by the conductivity data which showed poor surface conductivity of NaBH_4_ and ascorbic acid reduced samples due to formation of vacant spaces resulting in low connectivity of GO sheets.

The cross sections of the textile showed a complete non-bridged/non-covered layer signifying that the coating is only at the surface and it do not cover the cross section, Fig. 2f and g. This implies that the samples can suitably be used for e-textile applications without posing a potential threat for inherent sort circuiting as the two surface coated layers are well separated from each other. It was also observed that the spray coated sample had rGO layer more firmly attached to the textile surface compared to the brush coated samples (indicated by arrows in Fig. 2f). Poor penetration tendency of GO flakes in the brush coated samples can result in formation of a surface skin in these samples. The conductivity of the samples was retained after 10 washing cycles (samples were stirred under de-ionized water containing 1 wt% of Ethylan 1008, a non-ionic surfactant at 40 °C for 15 minutes and were washed under running water), Table S1 (ESI†) indicating that the coating had good wash fastness.

### Contact angle of GO coated textiles

Reduction of GO to rGO was expected to reduce the hydrophilicity of the coating due to removal of oxygen functionalities located on the edges and basal plane of the GO which can interact with water.^44^ The coating of rGO is expected to form a barrier for the penetration of water. Hence, the time of absorption can provide an indication on the uniformity of surface coating. The static contact angle of GO coated samples as function of the time was therefore studied and the values are listed in Table 2 and S2 (ESI†). Neat PET showed a contact angle of 110° ± 2 with time of adsorption of about 62 ± 4 s. Control GO coated textile (without reduction of GO), was very hydrophilic. It was difficult to measure its contact angle and time of adsorption because of rapid penetration of water. For reduced samples, the contact angle was directly proportional with the conductivity of the textiles. Thermally annealed samples with highest GO content showed the larger contact angle with highest time of absorption. PET samples reduced with NABH_4_ and ascorbic acid showed similar contact angle and time of absorption values which were lower than thermally annealed samples. This data agrees with the conductivity values reflecting poor surface connectivity of GO layers. Reduced form of GO is hydrophobic in nature and hence resulted in an increase in contact angle values. The results provided an indirect insight towards the conductivity of samples and the values of conductivity and contact angle complement each other on the uniformity of coating and the reduction efficacy. Thermally annealed GO samples without surfactants showed highest contact angle and time of penetration compared to the chemically reduced films.

### Mechanical properties

**Fig. 3:**
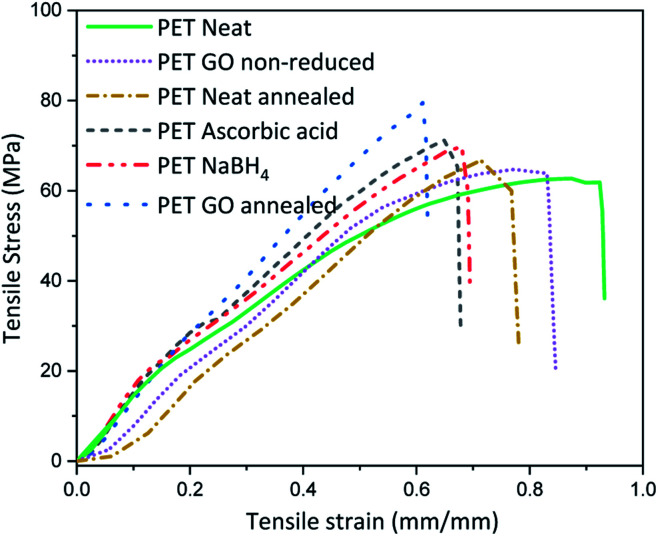
Tensile properties of GO coated samples.

Thermal or chemical reduction of GO coated textiles along with the additional impact of surface deposited GO can bring about alteration in the inherent mechanical properties. For this the mechanical properties of the samples with highest rGO content reduced *via* various approaches were analysed and the average stress–strain curves are plotted in Fig. 3 and Table S3 (ESI†). Neat PET shows maximum stress at 58 ± 6 MPa and breaking extension of around 90%. Coating of PET with 1.83% GO increases the stiffness by reducing the breaking extension to 10% and marginally increases the breaking modulus. This suggests that a coating of GO on neat PET fabric forms a film at the surface which in turn adds to the stiffness of the fabric, which could somewhat be expected knowing the outstanding mechanical properties of graphene. To check the influence of heating on the modulus and extension properties, neat PET was thermally annealed at 200 °C for 2 h and the breaking extension was found to decrease by ∼13% with slight increase in maximum modulus as compared to neat PET. Similarly, thermal annealing of PET above its *T*_g_ (∼70 °C, below the melting point of ∼260 °C) induces segmental mobility and results in close placement of individual fibers in absence of external stress to hold the structure. Inter fiber fusion occurring at this condition, can make the sample stiff which ultimately results in reduced extensibility and minor enhancement of breaking modulus. Thus, the breaking modulus of GO coated thermally annealed PET fabric (S5) was almost 25% higher than neat PET, with 40% lower breaking extension compared to neat PET. Synergistic effect of thermal annealing with GO coating is expected to bring about this increase in stiffness. The breaking stress of NaBH_4_ and ascorbic acid reduced samples *i.e.*, SN3 and SA5 were slightly higher than neat PET but almost similar to GO coated non-annealed. It was lower than that of thermally annealed PET samples, which indicates that annealing can bring about inter fiber fusion at spatial loci and enhance the stiffness of the PET fabric.

The abrasion resistance analysis of these coated samples was performed for 30 000 cycles on a Martindale abrasion resistance tester where we noticed a drop of surface conductivity over 10-fold magnitude. We are currently working to improve the abrasion resistance of these coatings *via* surface modification mechanism. However, these abrasion conditions are well beyond the standards.

## Conclusions

The present study focuses on development of uniform rGO coatings using a simple yet, energy efficient approach from dispersion of graphene oxide. Conventional dip coating was avoided to optimize usage of graphene and water. Spray coating resulted in the formation of a uniform deposition of GO onto the surface even in absence of any dispersing agent. Also, the surface coating of samples prepared using spray coating was found to be more strongly bound to the textile substrate compared to brush coated samples. The contact angle and the conductivity were found to be directly related to the efficacy of reduction and the continuity of coating. Hence the spray coated samples resulted in attaining highest conductivity, higher contact angle, high time of water penetration.

The reducing method also appeared to be central in the realization of conductive textile. Thermal annealing approach resulted in better conductivity compared to chemical reduction process of GO coated on textiles using sodium borohydride or ascorbic acid, as well as in a higher integrity of the resulting rGO layer. The structural defects induced by the wet nature of the chemical reduction turned out to impact negatively the mechanical properties of the coated PET. Even though the tensile modulus of the coated reduced samples was improved compared to neat textiles, it was for the thermally reduced rGO that the mechanical properties of PET were observed to be enhanced.

With this study, we demonstrate that adoption of the spray coating technique combined with a thermal annealing could be an efficient way to produce GO coated conductive textiles, while minimizing energy, and resources consumption.

## Conflicts of interest

There are no conflicts to declare.

## Supplementary Material

RA-010-C9RA09164E-s001
