# Supplementary material for: Conductive textiles prepared by spray coating of water-based graphene dispersions
Source: RSC Adv. 2020 Jan 13;10(4):2396–403. doi: 10.1039/c9ra09164e (PMC9048503; doi:10.1039/c9ra09164e)
Supplement: RA-010-C9RA09164E-s001 [file RA-010-C9RA09164E-s001.pdf]

Electronic Supplementary Material (ESI) for RSC Advances.
This journal is © The Royal Society of Chemistry 2019

Supplementary information

Conductive textiles prepared by spray coating of water-based graphene dispersions

Archana Samanta[a], Romain Bordes[a*]

[a] Department of Chemical Engineering, Chalmers University of Technology, Gothenburg, Sweden

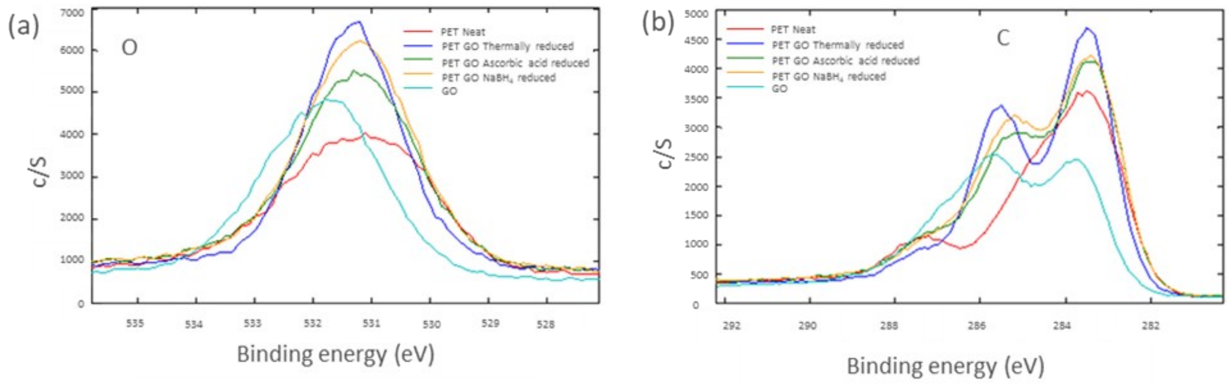

Figure S1. XPS data of samples. (a) high resolution oxygen peaks of samples. (b) high resolution carbon peaks of respective samples.

Table S1. Surface conductivity of samples after 10 washes.

Sample code	Surface conductivity (mS/m) Un-washed	Surface conductivity (mS/m) After 10 washes
SE1	--[c]	--[c]
SE2	--[c]	--[c]
SE3	0.31±.07	0.29±.09
SE4	0.37±.05	0.35±.05
SE5	0.45±.11	0.43±.07
S1	--[c]	--[c]
S2	0.37±.09	0.34±.10
S3	0.48±.10	0.46±.07
S4	0.69±.07	0.66±.05
S5	2.38±.11	2.16±.14
SA1	0.48±.11	0.46±.08
SA2	0.55±.08	0.53±.07
SA3	0.83±.09	0.79±.07
SN1	0.37±.06	0.35±.05
SN2	0.48±.08	0.45±.03
SN3	0.69±.07	0.66±.06
B1	0.41±.07	0.39±.05
B2	0.53±.04	0.50±.04
B3	1.28±.09	1.19±.07
PETN	--[c]	--[c]

[c] Surface resistance was too high to be measured by the instrument

Table S2. Contact angle values of rGO textiles.

Sl.No	Sample details	Contact angle
SE1	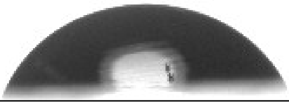	65°±11,
SE2	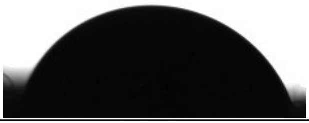	68°±7
SE3	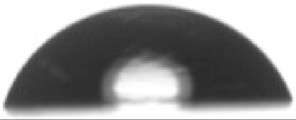	74°±8
SE4	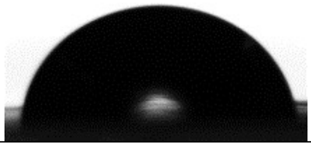	76°±10
SE5	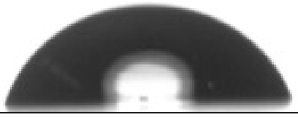	76°±14
S1	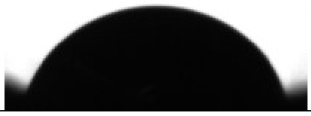	69°±7
S2	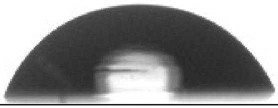	71°±8
S3	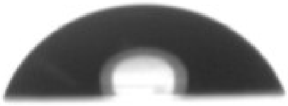	71°±3
S4	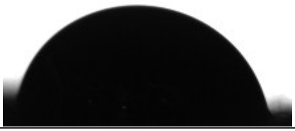	75°±2
S5	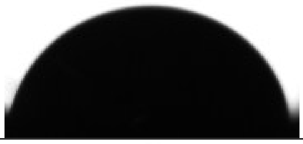	79°±8
SA1	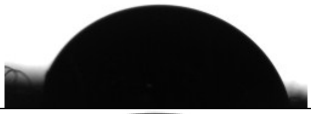	70°±5
SA2	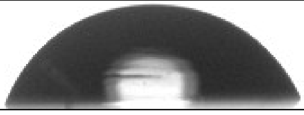	73°±4
SA3	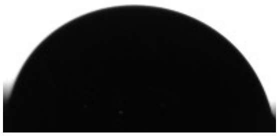	74°±2

SN1		68°±3
SN2	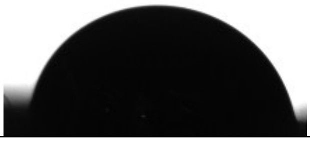	77°±3
SN3	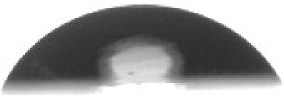	64°±4
B1	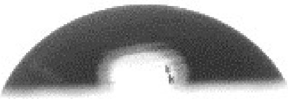	65°±8
B2	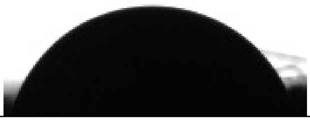	66°±5
B3	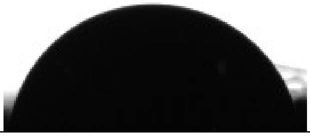	72°±7
PETN	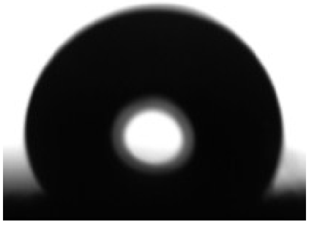	110°±2

Table S3. Tensile properties of GO coated samples

Sample	Tensile stress (Mpa)	Tensile strain (mm/mm)
PET neat	58±6	0.9±0.04
PET neat annealed	61±5	0.76±0.03
PET + GO	60±4	0.82±0.05
PET $NaBH_4$	68±5	0.70±0.04
PET ascorbic acid	71±4	0.66±0.03
PET GO annealed	79±5	0.54±0.02
